# 大剂量静脉铁剂治疗缺铁性贫血的应用进展

**DOI:** 10.3760/cma.j.issn.0253-2727.2022.11.014

**Published:** 2022-11

**Authors:** 立民 刘, 德沛 吴

**Affiliations:** 国家血液系统疾病临床医学研究中心，苏州大学附属第一医院血液科，江苏省血液研究所，国家卫生健康委员会血栓与止血重点实验室，苏州 215006 National Clinical Research Center for Hematologic Diseases, the First Affiliated Hospital of Soochow University, Jiangsu Institute of Hematology, Key Laboratory of Thrombosis and Hemostasis of Ministry of Health, Suzhou 215006, China

铁元素对于生命的维持发挥着至关重要的作用，它是几乎所有生命体不可或缺的物质之一。缺铁是指在明显缺铁性贫血之前的铁储备量减少或这一情况持续存在但没有进展。缺铁性贫血则是一种更严重的铁缺乏，为小细胞低色素性贫血[1]。缺铁及缺铁性贫血是目前全球范围内的健康问题之一，并带来了巨大的社会和经济负担。近年的研究显示，铁缺乏影响着全球超过20亿人的健康，全球范围内的缺铁性贫血病例超过了12亿[1]–[2]。常见的缺铁性贫血高危人群包括儿童、经期女性、孕妇、各类器质性疾病患者。有效地纠正缺铁和治疗缺铁性贫血是该疾病管理的重要问题之一。

当出现缺铁和（或）缺铁性贫血时应及时纠正和治疗。目前缺铁性贫血的治疗药物主要为口服和静脉铁剂。口服铁剂虽服用方便、价格低廉，但是其补铁效力不及静脉铁剂，而对于胃肠吸收障碍的人群则不利于纠正贫血。静脉铁剂较口服铁剂可更快补充铁元素以提升血红蛋白含量，改善贫血症状。但是既往的常规静脉铁剂存在不能一次性大剂量补充等问题。新型大剂量静脉铁剂已问世十余年，满足了临床上对于大剂量快速补铁的需求。然而，此方面的综合性分析报道在国内较少。本综述概括了缺铁性贫血的现状及治疗方案、静脉铁剂在疾病治疗中的应用以及大剂量静脉铁剂的研究进展，旨在为临床诊疗提供思路和依据。

一、缺铁性贫血现行的治疗方案

目前治疗缺铁性贫血的药物主要为口服和静脉铁剂。口服铁剂是纠正缺铁及治疗缺铁性贫血的主要药物。口服铁剂服用方便、价格低廉，但是对于胃肠疾病导致的吸收障碍等人群则不利于纠正贫血。口服铁剂补铁速度慢，纠正体内储存铁需要3～4个月。另外，口服铁剂可能存在一定的胃肠不良反应，以及增加患肠炎的风险[3]。既往研究中的上消化道内镜检查结果显示有16％的口服铁剂患者存在消化道黏膜铁沉积，并大多合并胃炎、甚至食管糜烂[4]。近年的研究发现口服铁剂可能影响肠道菌群，导致不利菌的繁殖与入侵，促进致病微生物（例如大肠杆菌）的生长，增加患肠道炎症的风险[5]。因此，口服铁剂为患者带来的益处尚不能一概而论，尤其对于胃肠疾病和胃肠切除术后的患者而言，应施以更有效及安全的补铁方案。

静脉铁剂不存在胃肠吸收的过程，因此可快速补充铁元素和提升血红蛋白水平，同时没有口服铁剂对胃肠道产生的不良反应和吸收障碍等问题。尤其对于围手术期及长期面临贫血风险的各类疾病的患者，静脉铁剂可更快满足患者的需求并减少输血量[3]。1932年，医学界开始使用含有羟基氧化铁的肠外补铁医治方法，但这种方法不良事件较严重[6]。1954年，肌肉注射右旋糖酐铁出现，但是这种给药方案造成患者肌肉疼痛并且需频繁注射[7]。静脉给药铁剂随后受到了更多关注，其问世产品亦不断更新。近十余年，新型静脉铁剂被全球近30亿人使用以治疗缺铁和缺铁性贫血，例如羧基麦芽糖铁和异麦芽糖酐铁[8]。对于长期贫血的患者如慢性肾病、炎症性肠病、产褥期人群等，大剂量静脉铁剂治疗是优选方案。因为大剂量静脉铁剂不但可以快速提升血红蛋白含量、缓解贫血症状，患者进行注射的频率低且血红蛋白浓度维持较好，不良反应少，大大降低了医疗成本[3]。

二、静脉铁剂药物

（一）已上市的静脉铁剂药物

已上市的静脉铁剂主要包括羧基麦芽糖铁、异麦芽糖酐铁、蔗糖铁、右旋糖酐铁、纳米氧化铁、葡萄糖酸钠铁等。静脉铁剂的使用剂量取决于治疗的目标是纠正贫血还是完全补足贮存铁；同时根据患者的体重、当前血红蛋白水平，以及每毫升铁剂中元素铁的含量来计算剂量[3]。此外，铁剂的选择亦受患者就诊频率的影响，无需频繁就医的患者可能更愿意选择大剂量静脉铁剂，例如羧基麦芽糖铁、异麦芽糖酐铁1000等[3],[8]。这些大剂量静脉铁剂通常一次性补充1 000 mg即可达到治疗目标，治疗效率高。

（二）国内外研究中的大剂量静脉铁剂药物

在我国的“药物临床试验登记与信息公示平台”以及国际的临床试验登记平台“ClinicalTrials.gov”上查询在研的静脉铁剂，以了解该方面的研究情况。在“药物临床试验登记与信息公示平台”中有1个检索结果，为《羧基麦芽糖铁注射液生物等效性研究》（CTR20220287）。在“ClinicalTrials.gov”中有188个初步结果（排除了暂停、终止、已完成、撤回的试验）。进一步查阅后发现在研药物仍以羧基麦芽糖铁、异麦芽糖酐铁、蔗糖铁为主，少数研究中是硫酸亚铁、氢氧化铁、右旋糖酐铁、柠檬酸焦磷酸铁、纳米氧化铁。从目前的研究来看，羧基麦芽糖铁、异麦芽糖酐铁、蔗糖铁等仍是被广泛应用的静脉铁剂。

（三）大剂量静脉铁剂在疾病治疗中的应用

我国《静脉铁剂应用中国专家共识（2019年版）》认为很多情况下静脉铁剂优于口服铁剂[3]，包括：（1）患者不耐受口服铁剂的不良反应；（2）患者更愿意通过1～2次就诊就补足贮存铁，而不愿耗时几个月；（3）持续性失血且超过了口服铁剂满足补铁需求的能力；（4）口服铁剂吸收不良的患者；（5）合并炎症而干扰铁代谢稳态；（6）预期失血量>500 ml的手术，或<6周内需行手术的铁缺乏患者。因此，静脉铁剂更适用于围手术期、妇产科、肾性贫血和炎症性肠病等人群。

1. 围手术期：围手术期的贫血较为常见。普通外科约有1/3的手术患者存在术前贫血，接受大手术的患者其术后贫血的发生率高达80％～90％[9]。髋、膝关节和股骨头置换术的患者术前贫血发生率为24％～43.9％，术后高达51％～89.1％[10]。心血管手术的患者由于手术部位特殊及体外循环的影响，患者大量失血等情况较为常见[11]。而围手术期的患者通常没有足够的时间通过口服铁剂来纠正缺铁和贫血，并且由于潜在的合并症，许多患者口服补铁治疗无反应。因此建议静脉铁剂用于治疗围手术期贫血。

在紧急手术、大出血或需要侵入性手术时，大剂量铁剂（1 000～1 500 mg）快速输注可恢复患者体内铁含量并改善术后预后[12]。近年研究表明静脉铁剂可显著改善手术患者术后的血红蛋白水平、使异体输血率降低了16％，并且不良事件较口服铁剂少[13]。对于新型静脉铁剂单次使用者，可予以大剂量输注以快速纠正贫血。这有助于减少并发症，促进患者术后恢复、降低术后感染、缩短住院时间并降低医疗费用。

2. 妇产科：妇产科患者及孕产妇是缺铁性贫血的高发人群。妇科患者术前贫血的患病率为24％～45％，妇科恶性肿瘤患者的贫血患病率高达81.4％[14]。孕期的铁需求量从1.5～2 mg/d增加至5～7 mg/d，而孕期缺铁影响着10％～90％的人群，这可能会影响胎儿的神经系统发育[8],[15]。此外，全球范围内的产后出血发病率为6％～11％，其中1％～3％为严重者；有34％的孕产妇因产后出血死亡[16]。因此建议铁缺乏的妇科围手术期患者和孕产妇进行补铁治疗，尤其是静脉铁剂的应用。

既往研究表明对于妊娠期、围产期和月经过多而致缺铁性贫血的人群，大剂量静脉铁剂较口服铁剂在患者血红蛋白反应、血红蛋白恢复至目标水平的时间以及减少不良事件方面更具有优势[17]–[21]。同时，大剂量静脉铁剂较口服铁剂更具良好的安全性，并且患者就诊次数少、医疗成本低、治疗依从性好[17],[19]。此外，近年的一项研究显示大剂量静脉铁剂羧基麦芽糖铁（体重<50 kg的患者使用500 mg，体重>50 kg的患者使用1 000 mg）和蔗糖铁（每次200 mg，每周最多600 mg）治疗妇科患者的术前贫血时，1 000 mg的羧基麦芽糖铁较蔗糖铁可快速纠正缺铁性贫血[17]。说明大剂量静脉铁剂用于妇产科治疗缺铁性贫血具有更好的有效性和安全性。

3. 肾性贫血：肾性贫血往往是非透析性慢性肾病（chronic kidney disease，CKD）患者常见的早期并发症，其主要原因之一是铁缺乏。NHANES调查显示CKD患者的贫血患病率为15.4％，高于非CKD患者的7.5％[22]。我国的研究显示，非透析CKD贫血的患病率高达51.5％，透析患者贫血患病率高达91.6％～98.2％[23]。但是非透析CKD患者只有27.1％接受了铁剂治疗，仅7.5％的患者接受了更有效和推荐的静脉铁剂治疗。积极治疗CKD贫血可延缓病程进展、改善预后，并且大剂量静脉铁剂可为患者带来更多益处。

FINDCKD研究是一项为期56周、开放标签、多中心、前瞻性、随机性的研究，涉及626例非透析依赖性且未接受促红细胞生成素的CKD贫血和铁缺乏的患者。研究发现与口服铁剂相比，接受大剂量静脉铁剂治疗的患者（在治疗的第4～48周中，每4周予500～1 000 mg铁剂）其血红蛋白水平可快速达标并维持在目标水平，并延迟和（或）减少了对其他贫血管理的需求。该研究未观察到静脉铁剂的肾毒性，表明其安全性亦良好[24]。此外，REPAIR-IDA试验亦证明大剂量静脉铁剂用于治疗CKD贫血具有良好的有效性和安全性，为患者带来更多获益[25]。

4. 炎症性肠病：铁缺乏在炎症性肠病（inflammatory bowel disease，IBD）中很常见，大约1/3的IBD患者伴有不同程度的贫血[8],[15]。对于IBD伴缺铁性贫血的患者而言，口服铁剂治疗存在较大的局限性，首先，患者胃肠道吸收受损、对口服药物耐受性差；再者，口服铁剂容易增加肠黏膜炎症，从而促进不良细菌的生长，对肠道微生物菌群造成不利影响。所以，静脉铁剂是这类患者优选的治疗方案，尤其对于IBD活动期的患者。

FERGIcor试验是一项随机、对照、开放标签、多中心的临床试验，招募了14个国家共计485例IBD伴缺铁性贫血的患者，以研究静脉铁剂治疗缺铁性贫血的有效性。该研究发现72.8％的羧基麦芽糖铁治疗的患者其血红蛋白提升至正常水平，高于接受蔗糖铁的患者（61.2％）。两种治疗方法在第12周时均改善了患者的生活质量，并且患者耐受性良好[26]。说明大剂量静脉铁剂应用于IBD伴缺铁性贫血的患者其有效性和安全性均较好。

5. 心力衰竭（心衰）患者：对于心衰的患者应评估其是否患有缺铁及贫血，因为缺铁可能影响线粒体功能从而导致心肌收缩异常，这亦影响着心衰的严重程度及预后。然而，铁缺乏和贫血在心衰患者中较常见。因此，2021年的欧洲心脏病学会急慢性心力衰竭诊治指南和2022年的美国心脏病学会/美国心脏协会临床实践指南均建议对于NYHA心功能Ⅱ～Ⅲ级的心衰伴缺铁患者，应用静脉铁剂有助于改善其心功能状态和生活质量。

近年的AFFIRM-AHF试验是一项关于大剂量静脉铁剂（羧基麦芽糖铁）对急性心衰发作后病情稳定的患者（伴缺铁）其结局影响的研究[27]。该研究中的患者来自欧洲、南美和新加坡的121个中心，其中大剂量静脉铁剂组558例，安慰剂组550例。给药剂量根据患者缺铁程度而定，治疗时间为24周。该研究证明对于缺铁、左心室射血分数低于50％且在急性心衰发作后稳定的患者，使用大剂量静脉铁剂治疗降低了心衰患者住院期间的风险，并且安全性良好[27]。更早的FAIR-HF试验发现，50％接受静脉铁剂治疗的心衰伴左心室射血分数降低且缺铁（无论有无贫血）的患者其症状有很大程度改善，而接受安慰剂者仅为28％[28]；静脉铁剂组有47％的患者在第24周时NYHA分级改善为Ⅰ或Ⅱ级，而安慰剂组为30％[28]。此外，EFFECTHF试验亦证明了大剂量静脉铁剂对于合并缺铁和缺铁性贫血的心衰患者，在改善其症状、心功能、生活质量等方面均具有良好的治疗效果[29]。

三、大剂量静脉铁剂较口服铁剂和二代静脉铁剂的优势

大剂量静脉铁剂近年发展迅速、应用广泛，例如羧基麦芽糖铁、异麦芽糖酐铁等；而二代静脉铁剂主要为小剂量给药的静脉铁，如蔗糖铁等。常见的大剂量静脉铁剂如Vifor公司研发的Ferinject^®^（羧基麦芽糖铁注射液），允许的单次最大剂量达1 000 mg，并且可快速给药（≤15 min）。此外，异麦芽糖酐铁亦是新型大剂量静脉铁剂的常用药物之一。

大剂量静脉铁剂较口服铁剂的优势突出体现于临床疗效上。从作用机制看，铁在胃肠道吸收的生物利用度低，仅有10％左右；所以对于一些患者口服铁治疗的效果差、耗时长，补足储存铁更是需要数月时间。对于CKD、IBD及其他处于慢性炎症状态的患者，由于铁调素升高从而抑制了口服铁在胃肠道的吸收，因此建议静脉铁剂治疗[3]。此外，对于胃肠疾病或术后患者，口服铁的吸收差；严重缺铁性贫血的孕妇，由于胎儿生长发育的巨大需求，口服铁剂的补铁效率也可能不太能满足临床需求[8]。另外，口服铁剂的胃肠道不良反应较明显，多数患者会因口服铁出现胃肠道反应，这影响了患者对治疗的耐受性和依从性。

大剂量静脉铁剂较小剂量给药方式的突出优势在于无需频繁给药即可达到理想的治疗效果，可减少患者去医院就诊的频率，从而减少对患者生活方式的干扰，因此患者的依从性更强[15]。大剂量静脉铁剂纠正贫血更快，更适合需要短期迅速提高血红蛋白的患者，比如近期要做手术的患者[3],[9],[15]。另外，目前的新型静脉铁剂由于其结构的创新完善，安全性更好，铁过载、超敏反应等不良反应发生率较低。

四、静脉铁剂和大剂量静脉铁剂的安全性良好

静脉铁剂具有良好的安全性，比口服铁剂不良反应更少。静脉铁剂的可能不良反应包括过敏反应、铁过载而致严重细菌感染的风险增加、药物外漏、低血压、肝损伤等[3]。但是严重的过敏反应和静脉铁剂带来的感染风险均较为罕见。其他严重不良反应亦不常见，并且静脉铁剂的胃肠道不良反应发生率更低[30]。一项荟萃分析纳入了103项与静脉铁剂有关的临床试验以分析静脉铁剂的安全性[30]。结果发现静脉铁剂不会增加严重不良事件的发生风险（*RR*＝1.04，95％ *CI* 0.93～1.17）；而静脉铁剂用于心衰患者时，严重不良事件的发生率降低（*RR*＝0.45，95％ *CI* 0.29～0.70）；与此同时，静脉铁剂导致感染的风险没有增加[30]。另有一项荟萃分析纳入了11项随机对照试验，比较了静脉铁剂与口服铁剂治疗妊娠期缺铁性贫血的情况；结果显示静脉铁不但可以快速提高血红蛋白含量，且静脉铁剂不良反应少（*OR*＝0.35，95％ *CI* 0.18～0.67）[31]。2018年的一项荟萃分析比较了大剂量静脉铁剂与低剂量静脉铁剂、口服铁剂，或无补铁治疗的透析患者的全因死亡率、感染、心血管事件发生或住院治疗的情况；同样显示了静脉铁剂、尤其是大剂量静脉铁剂用于透析患者亦具有良好的安全性[32]。因此，静脉铁剂因其有效性及安全性的优势被广泛接受和使用。

五、总结

大剂量静脉铁剂改善了临床上对缺铁性贫血的管理。静脉铁剂较口服铁剂可更快速补充铁储备、提高血红蛋白水平，并且不良反应小，总体安全性及耐受性良好。大剂量静脉铁剂适用范围广泛，对于围手术期、妇产科、炎症性肠病、慢性肾病贫血、合并铁缺乏的急慢性心衰患者等人群均是优选的治疗方案。患者接受大剂量静脉铁剂的治疗频率低、周期短，降低了就医负担，依从性更高。因此，大剂量静脉铁剂是治疗缺铁性贫血的优选方案之一，值得临床推广应用。
